# CMIC: an efficient quality score compressor with random access functionality

**DOI:** 10.1186/s12859-022-04837-1

**Published:** 2022-07-23

**Authors:** Hansen Chen, Jianhua Chen, Zhiwen Lu, Rongshu Wang

**Affiliations:** School of Information, Yunnan University, Chenggong Campus, Kunming, Yunnan China

**Keywords:** FASTQ, Quality score, Random access, Lossless compressor

## Abstract

**Background:**

Over the past few decades, the emergence and maturation of new technologies have substantially reduced the cost of genome sequencing. As a result, the amount of genomic data that needs to be stored and transmitted has grown exponentially. For the standard sequencing data format, FASTQ, compression of the quality score is a key and difficult aspect of FASTQ file compression. Throughout the literature, we found that the majority of the current quality score compression methods do not support random access. Based on the above consideration, it is reasonable to investigate a lossless quality score compressor with a high compression rate, a fast compression and decompression speed, and support for random access.

**Results:**

In this paper, we propose CMIC, an adaptive and random access supported compressor for lossless compression of quality score sequences. CMIC is an acronym of the four steps (classification, mapping, indexing and compression) in the paper. Its framework consists of the following four parts: classification, mapping, indexing, and compression. The experimental results show that our compressor has good performance in terms of compression rates on all the tested datasets. The file sizes are reduced by up to 21.91% when compared with LCQS. In terms of compression speed, CMIC is better than all other compressors on most of the tested cases. In terms of random access speed, the CMIC is faster than the LCQS, which provides a random access function for compressed quality scores.

**Conclusions:**

CMIC is a compressor that is especially designed for quality score sequences, which has good performance in terms of compression rate, compression speed, decompression speed, and random access speed. The CMIC can be obtained in the following way: https://github.com/Humonex/Cmic.

**Supplementary Information:**

The online version contains supplementary material available at 10.1186/s12859-022-04837-1.

## Background

Modern DNA sequencing technology has generated a large amount of genomic data, and with the emergence and maturity of NGS (next-generation sequencing) methods, genome sequencing is becoming less expensive. In 2014, the cost of sequencing a human genome was limited to $1000 [1], and the lower cost means that there will be an explosion of genomic data. Today, the latest systems can sequence the equivalent of 48 complete human genomes at once at 30× coverage. These genomes produced approximately 6 TB of FASTQ files [2]. Such large-scale sequencing creates challenges for data storage, transmission, and analysis. Therefore, it is necessary for us to develop a genome sequence compressor with high efficiency.

**Fig. 1:**
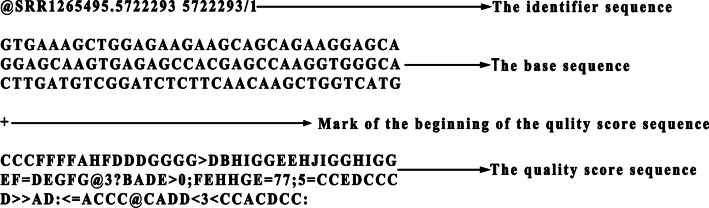
The example of FASTQ format

The output of modern DNA sequencing is typically stored in FASTQ format [3], as shown in Fig. 1, which is a file format containing the following types of data:

1. The identifier sequence contains the ID of the reads. It begins with @ followed by an optional description.
2. The base sequence contains bases of a read. It usually has four kinds of bases (A, C, G, T).
3. The quality score sequence contains quality score symbols of a read. It reflects the level of confidence in the readout of each base. Its specific calculation formula is $S = - 10\log_{10} P$, where $P$ is a reference sequencing error probability. Quality values range from 33 to 73 (or from 64 to 104) and are represented by ASCII symbols.

For each read, there is a paired line of scores that is equal in length to the read length. The quality score information is always of the same length as the respective base sequence. Some FASTQ files have a fixed read length, and the length of the quality score lines are also fixed; others have variable read lengths, and therefore, the lengths of the quality score lines are also variable.

Among FASTQ files, the base sequence is highly redundant, with only four possible values of ATCG in the sequence. The quality score sequence is much more difficult to compress than the base sequence due to low redundancy and the fact that there are as many as 71 possible values in the sequence. Additionally, more values in the quality scores also lead to the compression being less efficient. Since the final compression step in the proposed method is completed by ZPAQ, which is a statistical model-based algorithm, a larger alphabet size means more difficulty in learning the context model for a given source sequence, and thus, the compression efficiency is affected. Moreover, in the lossless FASTQ compressed files, the quality scores account for approximately 70% [4]. Therefore, the need for an algorithm that can efficiently and losslessly compress quality scores has become critical. In addition, the quality score is important for mutation testing. When retrieving a list of specific protein sequences, we only use a part of the genome data [5], which means that only some of the bases and their corresponding quality scores in a FASTQ file need to be retrieved, and the need for random access functionality on the compressed genome data arises. Recently, the biotechnology industry has made substantial progress, and the compression of genomic sequences is close to practical application. However, most previous genome data storage efforts do not have random accessibility, which means that they have to decode the entire compressed file to retrieve a specific segment in a sequence [6]. Since quality scores consist of an important part of a FASTQ file, an efficient compression algorithm for them should also implement random access to the compressed quality score information related to a specific base sequence. Obviously, if we want to improve the random access efficiency, we need to divide the genome sequence into blocks, but this requires an appropriate indexing method to quickly find the required quality score sequence and minimize the impact of the index file on the final compression efficiency. However, the block size directly affects the efficiency of compression and random access, so we need to achieve a reasonable balance between the two.

Most current quality score compressors use run-length encoding [7] or build complex context models for compression, and they do not support random access. The advantage of using run-length encoding is that the compression speed is fast; however, the compression ratio is relatively low, and the robustness is poor (compression may not perform well for different types of FASTQ files). The benefit of using complex context modeling for compressors is the high compression ratio, but the compression speed is slower than the other compression methods (Conversely, some compressors try to increase the compression speed by building simple probabilistic models, but the compression ratios of these methods are usually poor). It can be seen that the compression speed and the compression ratio are not independent and are mutually constrained. Most compressors weaken one to enhance the other. By improving the parallelism of the algorithm and use more CPU cores, we can improve the compression ratio and speed at the same time.

In this paper, we propose a new quality score compressor, CMIC. To improve the performance of CMIC, we made the following contributions:

1. We propose a new mapping method that makes full use of the correlation between adjacent quality scores and improves the efficiency of context modeling entropy encoding.
2. By building a highly effective light weight index, it implements and speeds up the random access.

This article is organized as follows. The second section mainly describes the related work. The third section introduces the specific implementation method of CMIC. The fourth section discusses the experimental results. In the fifth section, we provide the conclusion and the directions of future work.

To date, quality score compressors can be divided into two categories: lossy and lossless approaches. Compared with the lossless compressor, a lossy compressor can considerably improve the compression efficiency, but it is still controversial in practical applications [8]. Because the quality score represents the quality of each base, the value of the quality score shows the potential error probability of the base. The larger the value is, the smaller the possibility of a sequencing error. Therefore, improper processing may lead to errors in the analysis results of the gene data.

With regard to lossy quality score compressors, in 2016, the MPEG standardization committee proposed a framework to measure the impact on variant calling for human genomes [9]. However, long before the previous framework, the lossy quality score compression tools QVZ [10], R-Block and P-Block [11] could not only maintain variant calling performance but even improve it in some cases. However, considering that the lossy quality score compression technology is still controversial, this paper focuses on solving the related problems of lossless quality score compression.

In terms of lossless quality score compression, Fqzcomp [4] uses a hybrid statistical model to compress the sequence and quality scores: the identifier sequence is encoded by delta coding after preprocessing, and the base sequence is compressed based on a limited context model. For DSRC2 [12], when compressing the identifier sequence, the same method has been used as the one in Fqzcomp. Huffman encoding is used for base sequence compression. When compressing the quality scores, mixed encoding (Run-Length and Huffman encoding) is used. To date, many professional FASTQ compressors still use the above methods to deal with quality scores. For example, LW-FQzip2 [13] preprocesses the quality scores using run-length encoding and then uses the combination of the PPM model and the arithmetic encoder to compress the preprocessed data. LFQC [14] divides the quality score file into several parts. It can easily determine whether run-length encoding or Huffman encoding will be used by checking the average run length of each quality score sequence. In addition to the above compression algorithms, there are some general solutions, such as Gzip [15] and 7-Zip [16]. Most of the public repositories still use them to store FASTQ files due to their simplicity.

Of course, with the continuous improvement of the sequencing speed, some professional quality score compressors have also been developed, such as SCALCE [17]. The algorithm uses the core substring as the similarity measure to group similar sequencing short fragments. These core substrings are generated by LCP (locally consistent parsing) [18–20]. A third-order context model is used for the final compression. In fact, as early as 2016, the CSAM [21] algorithm realized the random access function on SAM files. It could process SAM data without decoding the whole compressed file. The AQUa [8] algorithm is a quality score compressor based on the AFRESh [22] framework. The authors develop and integrate four new encoding tools to make better use of the redundancy in the quality scores and then use the Context-Adaptive Binary Arithmetic Coder to compress the quality scores. CABAC is a tool for lossless entropy coding. It is widely used in the field of compression [23, 24]. At the same time, AQUa supports random access to quality scores. In addition, the LCQS [25] algorithm uses a general compressor libzpaq [26] to compress quality scores. By optimizing the source code, the running speed of the algorithm is greatly improved. LCQS uses the packing algorithm to make use of the correlation among the adjacent quality scores. However, they thought that the quality score with the most occurrence was the score with the largest value, so their packing rules only considered part of the combinations of the neighboring quality scores. Nevertheless, the score with the most occurrence is in fact not the one with the largest value. The newly proposed FCLQC [27] algorithm uses concurrent programming to greatly improve the running speed of the algorithm, which shows at least 31× of the compression speed improvement, but the compression efficiency is reduced on average by 8.62%.

Through the description of the above algorithms, we find that it is difficult to implement the function of random access if we use the method of context modeling arithmetic encoding for the whole quality score sequence. For the Run-Length and Huffman encoding technology, the compression efficiency is greatly related to the characteristics of the quality scores, and the robustness of the algorithm is generally not satisfactory. In summary, we are interested in designing an algorithm that satisfies the following:

1. Context modeling arithmetic encoding is used to improve the compression ratio, but we want to directly use the correlation of adjacent quality scores to improve the modeling efficiency. At the same time, the adaptive context model is used to improve the robustness of the algorithm for different genome sequences.
2. The function of random access on compressed files is implemented; in other words, a specific target quality score line can be accessed without decompressing the whole compressed file (only part of the file is decompressed).

## Implementation

The framework of the CMIC algorithm is as follows. First, we classify the quality score lines in different reads into various categories according to their statistical characteristics. The objective of this step is to group together the quality score lines with similar statistical characteristics to improve the statistical accuracy of context modeling. Next, CMIC performs the mapping operation on quality scores. Mapping directly utilizes the correlation between the adjacent quality scores (since there are few abrupt changes in adjacent quality scores) and improves the efficiency of context modeling in the compression phase. We use the idea of run length encoding to map multiple quality scores into one symbol. The advantage of this operation is that it improves the efficiency of describing the neighboring quality scores with similar values and greatly speeds up the context model-based compression. Then, we propose a lightweight index building method to support the random access function. We consider both indexing speed and the index directory size to keep the size as small as possible, while the random access efficiency is not apparently affected. Finally, the algorithm uses the general compression library libzpaq, and we use the parallelism of the library to compress multiple blocks at the same time to improve the compression speed.

### Classification

We can find that in a FASTQ file, the content of quality score lines can be very different. Many compression algorithms use this feature, such as CARGO [28], which first analyzes and rearranges the data, then aggregates the quality scores with similar properties, and finally compresses the file. In the AQUa algorithm, the authors propose five different coding tools, and select the most efficient coding tools for different quality score lines. The main reasons for classifying the quality score lines are as follows:

1. Classification can improve the robustness of the compressor, and get better compression results for different FASTQ files.
2. Classification can improve the efficiency of context modeling and improve the compression speed.

The efficiency of context modeling is related to the distribution of data, and data with poor statistical characteristics will affect the modeling of other data. Therefore, we want to separate the quality score lines with different statistical characteristics. Through the classification algorithm, we classify the quality score lines with good statistical characteristics into one class, which can effectively improve the efficiency of context modeling. The other is the quality score line with poor statistical characteristics. Although the efficiency of data context modeling of this class is poor, it does not affect the compression of the other class.

In this paper, we present a statistics-based quality score classification algorithm. It is well-known that before the base sequence is compressed, the bases are usually divided into K-mers. The K-mer contains high-order context information, which can be applied to the compression of quality score lines. We assume that the quality line is $r$, which is a string consisting of $\left| r \right|$ quality scores. Taking a string $\beta_{k}^{i}$ of length $k$ in $r$ starting from the $i$ th position, $\beta_{k}^{i} = r\left[ i \right]r\left[ {i + 1\left] { \cdots r} \right[i + k - 1} \right]$,$0 \le i \le \left| r \right| - k + 1$, $\beta_{k}^{i}$ is referred to as a “K-mer” in $r$. We assume that the sample distribution in a FASTQ file is consistent with the overall distribution. To speed up the classification, we take some quality score lines as the sample (usually the first M lines of the file). We classify each quality score line according to the number of K-mer repetitions in the sample. Suppose there are $J$ distinct types of K-mers in the sample; we count the number of their occurrences through $N_{K}^{j} , \left( {j = 1, \ldots ,J} \right)$. If the number of all possible K-mers in $r$ is $N_{K}^{{}}$, we can define $\overline{q}_{k}^{j} = \frac{{N_{K}^{j} }}{{N_{K}^{{}} }}$, which reflects the proportion of the *j*th K-mer in all K-mers in the sample. Let $\overline{{e_{l} }} = \mathop \sum \limits_{s} \overline{q}_{k}^{s} ,\left( {l = 1, \ldots ,M;s = 1, \ldots ,S_{l} } \right)$, where $S_{l}$ is the number of different K-mers in the $l$ th quality score line, and $\overline{{e_{l} }}$ is the sum of proportions of different K-mers in the $l$ th quality score line. Evidently, if all the different K-mers in the $l$ th quality score line are repeated more times in the sample, the value of $\overline{{e_{l} }}$ will be larger and vice versa. We group the quality score lines that have larger $\overline{{e_{l} }}$ values, which is advantageous for the entropy encoder based on context modeling to compress quality score lines with K-mers repeated more times. Then, we can find the maximum value of $\overline{{e_{l} }}$ within the sample by $L_{m} = \mathop {\max }\limits_{l} \overline{{e_{l} }}$. Finally, $L_{m}$ is used to normalize $\overline{{e_{l} }}$ for all the quality score lines. In this way, we can use $Mx$ as the classification feature for the quality score lines ($Mx = \frac{{\overline{{e_{l} }} }}{{L_{m} }}$). Usually, $Mx$ is a real number between 0 and 1. Because $L_{m}$ is obtained for the sample, $Mx$ could be larger than 1 for some quality score lines out of the sample; at this point, we set the value of $Mx$ to 1 (the classification standard is described later).

To facilitate our mapping and indexing, we divide the quality score lines into two categories based on $Mx$, which is obtained after preprocessing (class A and class B). The classification process is as follows:

1. The statistical information $\beta_{k}^{i}$ of the first M lines in the sample is collected.
2. $N_{K}^{j}$ and $\overline{q}_{k}^{j}$ are calculated for all distinct K-mers in the sample.
3. $\overline{{e_{l} }}$ is calculated for each quality score line in the sample.

When $L_{m}$ is obtained, $Mx$ can be calculated. According to a threshold $\alpha$, the quality score lines are divided into two categories, depending on whether $Mx$ is greater than $\alpha$.

### Mapping

The quality scores are saved in FASTQ files with 8-bit ASCII encoding. They usually show the following characteristic: in a quality score line, some quality scores repeat a lot, and adjacent quality scores are similar. This means that in a quality score line, quality scores change slowly in most cases. This characteristic makes quality score lines suitable for compression using a method similar to run-length encoding, and it is reasonable to map some adjacent quality scores into one character. Next, the mapped string is statistically modeled and entropy encoded. In this way, the correlation among the adjacent quality scores can be used directly. Many professional FASTQ compressors adopt the above methods, such as DSRC2, Fqzcomp and LFQC. These methods achieve good compression ratios and compression speeds. The number of encoded characters is reduced by mapping. The compression speed can be improved.

Specifically, we use a one-to-one rule to map more than one original score with values from 33 ~ 104 to one symbol with values from 1 ~ 512. The basic principle of mapping is to represent the substring with a large number of repetitions with a single character. We calculate a quality score C that appears most frequently in quality score lines after classification and then determine the mapping rules according to C. This shows that the possibility of a continuous occurrence of C is high, and C can be combined with other quality scores as much as possible. The more quality scores that satisfy the mapping conditions, the more quality scores can be combined to reduce the number of scores. Once we obtain C, we map the quality score with the value that is close or equal to C.

In LCQS, they only consider the case that the adjacent quality scores are less than or equal to C. In some FASTQ files, the most frequent occurrence of C is indeed the largest quality score, but the distribution of the quality scores in many FASTQ files is uncertain. This means that C can be an intermediate value among quality scores, which has a great negative impact on the compression results. In some extreme cases, the minimum quality score is C, and there is no mapping for the quality scores.

Therefore, we design the following quality score mapping rules:

Assume that $q_{i}$ is the $i$ th quality score in a quality score line.

1. When the values of the adjacent quality scores are equal to C, the next quality score $q_{i + k} (k \le 55$) is investigated until $q_{i + k}$ is no longer equal to C, we record the value of $k$, and map $q_{i} q_{i + 1} q_{i + 2} \ldots q_{i + k - 1}$ to a symbol with value $\left( {k + 200} \right)$.
2. When the value of the adjacent quality score is not equal to C, it can be divided into the following situations:
  - First, we discuss the case when the adjacent quality scores are close to C and belong to [C-3, C + 3]. At this time, at most three adjacent quality scores can be mapped to one character. If $q_{i} q_{i + 1}$ belong to [C, C + 3], then they are mapped to (($\left( {C + 7} \right) - q_{i} )*8 + \left( {\left( {C + 7} \right) - q_{i + 1} } \right) + 255)$; if $q_{i} q_{i + 1}$ belong to [C-3, C], then they are mapped to (($q_{i} - \left( {c - 7} \right)*8 + \left( {q_{i + 1} - \left( {c - 7} \right) + 73} \right)$. If $q_{i} q_{i + 1} q_{i + 2}$ all belong to [C, C + 3], then they are mapped to (($\left( {C + 3} \right) - q_{i} )*16 + \left( {\left( {\left( {C + 3} \right) - q_{i + 1} } \right)} \right)*4 + \left( {\left( {C + 3) - q_{i + 2} } \right) + 319} \right)$; If $q_{i} q_{i + 1} q_{i + 2}$ all belong to [C-3, C], then they are mapped to (($q_{i} - \left( {C - 3} \right))*16 + (\left( {q_{i + 1} - \left( {C - 3} \right)} \right)*4 + \left( {\left( {q_{i + 2} - \left( {C - 3} \right)} \right) + 137} \right)$; If one of $q_{i} q_{i + 1}$ is a member of [C, C + 3] and the other is a member of [C, C + 7], then they are mapped to (($\left( {C + 7} \right) - q_{i} )*8 + \left( {\left( {C + 7} \right) - q_{i + 1} } \right) + 255)$; If one of $q_{i} q_{i + 1}$ is a member of [C-3, C] and the other is a member of [C-7, C], then they are mapped to (($q_{i} - \left( {c - 7} \right)*8 + \left( {q_{i + 1} - \left( {c - 7} \right) + 73} \right)$.
  - When the adjacent quality scores are slightly far from C, they do not completely fall within [C-3, C + 3] but still fall within [C-7, C + 7]. At this time, two adjacent quality scores can be mapped to one symbol. We check $q_{i + 1}$. If $q_{i} q_{i + 1}$ belong to [C, C + 7], we map them to (($\left( {C + 7} \right) - q_{i} )*8 + \left( {\left( {C + 7} \right) - q_{i + 1} } \right) + 255)$; if $q_{i} q_{i + 1}$ all belong to [C-7, C], we map them to (($q_{i} - \left( {c - 7} \right)*8 + \left( {q_{i + 1} - \left( {c - 7} \right) + 73} \right)$.
3. If the current quality score $q_{i}$ is far from C, it is obvious that none of the above conditions is true. We directly map $q_{i}$ to $(q_{i} - 32)$ and continue to check the next quality score until the end of the quality score line.

By setting appropriate mapping rules, we consider all the cases of quality scores, and the algorithm is greatly improved and can be more adaptable to different types of FASTQ files. For a better understanding of the algorithm, please refer to the pseudocode shown in Additional file 1.

### Random access and indexing

**Fig. 2:**
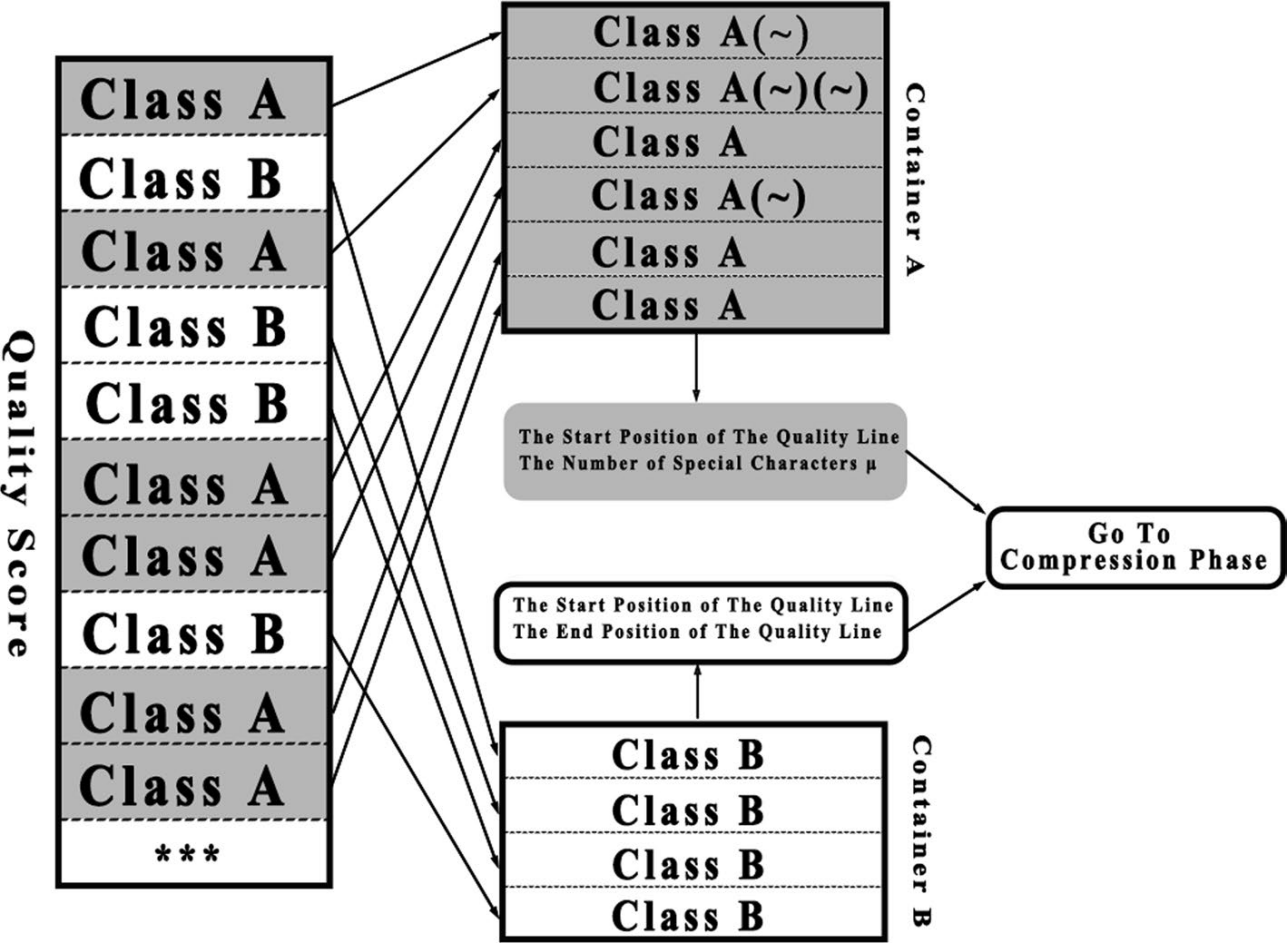
The Structure of the random access and index

After the classification step, quality score lines are divided into class A and class B, as shown in Fig. 2. To support random access, we have to combine these two types of quality score lines into data blocks for the final compression. In this way, the data blocks can be decompressed separately to implement random access. Because it is of little importance to study a specific quality score, the focus of our algorithm is allowing users to randomly access quality score lines in a certain range.

We set up two containers, A and B (gray and white parts in Fig. 2). Container A stores the quality score lines of class A with more K-mers repeated frequently to obtain better statistical information and better compression efficiency in the final compression process. We check over the quality score file; if a quality score line is of class A, then its content is added to container A. When a class B line appears, we replace the line with a special character (a character that does not exist in the quality score line) and add that character to container A as well. If there are consecutively occurring quality score lines of class B, we add the corresponding number of special characters to container A. In this way, the order of the quality score lines in container A is consistent with the order of the lines in the original file. However, the contents of the class B quality score lines are not reserved in container A, and thus, container B has to be used to save the contents of the class B quality score lines. Therefore, when a quality score line belonging to class B is found, we first add a special character to replace the line in container A, and then add the content of this class B quality score line to container B. Container B stores the quality score lines with K-mers less frequently repeated. When container A or B is full, the content of the container is sent to the compressor as a data block for compression.

In AQUa, to allow random access, the algorithm must create additional entry points for CABAC, where the byte addresses of the random access points are stored in a separate XML file. As a result, AQUa requires a large index file, and the maximum overhead of the index files is close to 3.5%, which affects the compression efficiency. At the same time, because AQUa cannot compress all kinds of FASTQ files, random access cannot be applied to some datasets (for example, the size of the read length is not fixed).

In CMIC, because quality score lines are stored separately in data blocks of classes A and B, to improve the efficiency of random access, we also need to build an index file to quickly find a specified quality score line. At the same time, to avoid the defects of AQUa, we create a lightweight index and reduce the size of the index file to improve the final compression ratio.

We need to build index tables for A and B (the rounded rectangle in Fig. 2). For container A, the index table stores the start position $S_{i}^{A}$ of the quality score line in container A and the number of special characters $\mu$. By querying $S_{i}^{A}$, we can quickly find the data block where the target sequence is located because the special characters in container A represent the position information of the class B quality score lines. Parameter $\mu$ can help us calculate the end position of the quality score line in container A and the positions of the class B quality score lines in container A. For container B, the index table stores the starting position $S_{j}^{B}$ and the ending position $E_{j}^{B}$ of the quality score line in container B. By querying the information of $S_{j}^{B}$ and $E_{j}^{B}$, we can rapidly find the data block where the target quality score line is located. The advantage of building a lightweight index is that the size of index files is greatly reduced, while the disadvantage is that a searching operation is needed during the random access process, but it only takes a small amount of time compared with the whole process.

Suppose the user wants to find the quality score lines within the range [a, b] ($a,b$ are the quality score line numbers in the quality score file). We first query the index table of container A and locate the target lines in a range of class A data blocks. Thus, we can obtain all class A lines in $\left[ {a,b} \right]$. For class B lines, the special character stored in container A and the number information in $\mu$ record its location information. The start line $a_{B}$ and the end line $b_{B}$ of class B in $\left[ {a,b} \right]$ are calculated by the information in the index tables:

Assume the number of quality score lines of class A before $a_{B}$ is $N_{a}$, $a_{B} = N_{a} + S_{i}^{A}$. When we find the $\mu$ th special character, it indicates that this position represents the last class B line in class A data blocks. Assume the number of quality score lines of class A before $b_{B}$ is $N_{b}$, $b_{B} = N_{b} + \mu + S_{i}^{A}$. Then, we query the index table of container B and locate $\left[ {a_{B} ,b_{B} } \right]$ in class B data blocks. Finally, the decompression operation is performed to complete the random access.

Since the index table is ordered, we can use the binary search algorithm to query the index table. Typically, we need to traverse the index table from scratch to locate a block of data. The binary search can effectively reduce the search time. The “Go to Compression Phase” block in Fig. 2 means that the algorithm goes to the compression phase after the index is established. In addition to extracting a specific range of quality score lines through random access, when we decompress the entire file, the order of the decompressed quality score lines is the same as their original order.

### Compression

In the last step of compression, we use libzpaq, an open-source compression library, which is the most advanced back-end compression algorithm. Although libzpaq has high compression performance, its compression speed is very slow. Therefore, optimizing libzpaq code is the focus of this step. In recent years, with the continuous update of the version, the compression speed of libzpaq has been substantially improved.

We combine the characteristics of libzpaq and CMIC (we adopt the strategy of block compression). The algorithm realizes the parallelization of the compression process by using SIMD (single instruction multiple data) technology. Libzpaq has a JIT version and a non-JIT version, and the JIT version is used in CMIC. The advantage of using this strategy is that we can make full use of the advantages of multiple CPU cores in parallel computing and greatly improve the compression speed. Using more CPU cores will improve the compression speed. However, the whole file is segmented and compressed by different threads; then, the compression ratio will be affected, and the CPU and memory occupation running the algorithm is large. However, with the rapid development of hardware, we are no longer limited to the slow speed and the small memory computers of the past. Therefore, we should try to maximize the performance of our algorithm.

## Results

In this section, we describe the experimental configuration in details and verify the superiority of CIMC compared with other compressors, mainly in terms of the following aspects: robustness, compression ratio, compression speed, random access speed, and decompression speed.

### Datasets and compared algorithms

In order to ensure that the datasets are fair and reasonable, we have selected some genomic datasets in the MPEG HTS compression working group. On the one hand, with huge demand and rapid development of genomic information compression, the standardization of genomic data benchmark has become an important task. The MPEG HTS compression working group is aware of the need and is building up the genomic data benchmark. They have done a better job on this side, which is the basis for our selection of test data. On the other hand, in order to make the experimental results more accurate, we should pay attention to the selection of test data involving different species, different platforms, different read lengths, and different sizes. Based on the above criteria, we selected the datasets shown in Table 1. They are publicly available from https://trace.ncbi.nlm.nih.gov/Traces/sra/.

**Table 1:** Detailed descriptions of tested genome datasets

Code	Datasets	Platforms	Organism	Bases (Mbp)	Read length	Size (Quality Score)
1	SRR1284073	PacBio	Escherichia coli	649.4	(130,10,000)	476,930,701 bytes
2	SRR327342	Illumina	S.Cerevisiae	2100	75	2,105,137,860 bytes
3	SRR870667	Illumina	T.Cacao	12,600	74 or 108	11,455,676,056 bytes
4	ERR091571	Illumina	Homo sapiens	42,700	100	43,133,335,476 bytes
5	SRR003187	LS454	Homo sapiens	803	(500,1000)	798,985,944 bytes
6	SRR003177	LS454	Homo sapiens	855	(500,1000)	850,464,554 bytes
7	SRR007215	ABI Solid	Homo sapiens	238.6	25	248,099,332 bytes
8	SRR010712	ABI Solid	Homo sapiens	431.6	35	443,972,736 bytes
9	SRR070253	ABI Solid	Homo sapiens	45,600	50	12,719,021,580 bytes
10	SRR801793	Illumina	Legionella pneumophila	1100	100	1,092,105,122 bytes
11	SRR14340293	OXFORD NANOPORE	Puccinia graminis	8900	(1000,10,000)	7,782,970,748 bytes

All tests are performed on a server with Intel Xeon(R) Gold 6240 CPU(2.60 GHz *72) with 251.3 GB of memory, running the CentOS Linux 7 operating system.

To better evaluate the performance of CIMC, we chose two types of lossless compressors, the first type is the general purpose lossless compressor, the second is the professional quality score lossless compressor. We chose LCQS, AQUa and FCLQC as the professional quality score compressors for comparison. For general lossless compressors, we choose 7-Zip and Gzip for comparison.

### Experimental results

The experimental results are shown in Table 2. As we can see, in terms of the compression rate, we achieve the best results for all datasets compared with other compressors (Because FCLQC [19] focused on improving the compression speed, so the compression rate is not very ideal, we did not add it to the control group). The compression rate is the value of bits per quality score. The best experimental result is the No.9 dataset with a compression rate of 1.01. In particular, compared with LCQS, 7-Zip, Gzip, the file size is reduced by up to 30.65%, 59.07%, and 94.05% respectively for the No.5 dataset using CMIC. Compared with AQUa, we can also see better compression rates for all of the datasets, with file sizes being 18.78% ~ 28.31% smaller than AQUa. At the same time, AQUa is not good at compressing FASTQ files with variable read lengths. CMIC is apparently effective both in terms of the compression rate and robustness.

**Table 2:** Comparison results of compression rates

Dataset	Compression rates (bits per quality score)					CMIC File Size versus			
	CMIC	LCQS	AQUa	7-zip	Gzip	LCQS	AQUa	7-zip	Gzip
1	2.10	2.27	–	2.54	2.79	− 8.13%	−	− 20.99%	− 33.17%
2	2.75	3.05	3.38	3.37	3.74	− 10.71%	− 22.78%	− 22.46%	− 36.03%
3	2.31	2.38	–	2.85	3.05	− 2.96%	−	− 23.42%	− 31.95%
4	2.01	2.04	2.52	2.44	2.86	− 1.49%	− 25.64%	− 21.82%	− 42.51%
5	1.33	1.74	–	2.12	2.59	− **30.65%**	–	− **59.07%**	− **94.05%**
6	1.38	1.70	–	2.08	2.54	− 23.34%	–	− 50.52%	− 83.91%
7	4.13	4.68	4.91	5.09	5.26	− 13.35%	− 18.78%	− 23.17%	− 27.17%
8	4.20	4.75	5.02	5.15	5.32	− 13.14%	− 19.73%	− 22.65%	− 26.67%
9	1.01	1.16	1.30	1.28	1.37	− 15.08%	− **28.31%**	− 26.43%	− 35.45%
10	2.47	2.50	3.02	2.96	3.44	− 1.05%	− 22.32%	− 19.96%	− 39.17%
11	3.14	3.82	–	4.23	4.52	− 21.91%	−	− 34.95%	− 44.03%

Bold denotes the best compression rates for compressors

**Fig. 3:**
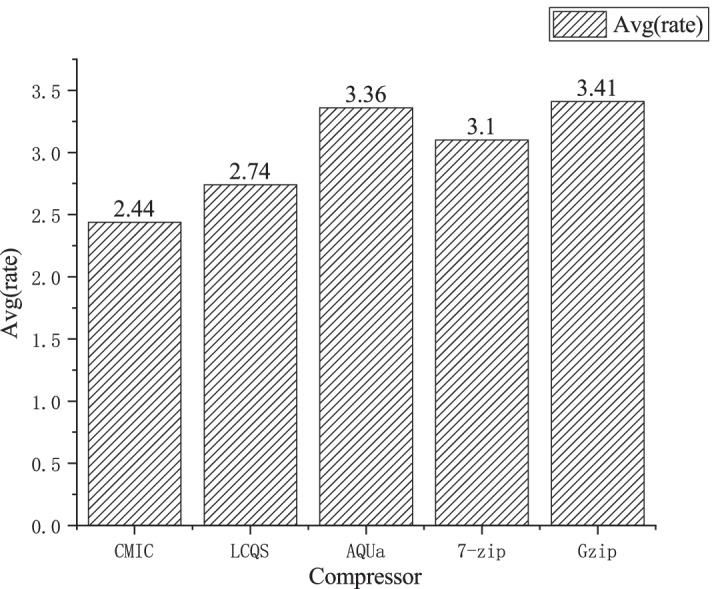
The averaged compression rates of five compressors over 11 datasets

Figure 3 shows the average compression rates of the five compressors over the datasets, and it is clear to see that the proposed algorithm has the best averaged compression performance.

Through observation on Tables 1 and 2, we have found that the compression rates of CIMC have a strong relationship with the read lengths of FASTQ files. The smaller the read length, the lower the compression rate. Generally speaking, for the same read length, the larger the dataset is, the better the compression performance is.

Table 3 shows the results of the compression speed of the CMIC compared with other four compressors. Since AQUA is unable to process FASTQ files with variable read lengths, so we do not use AQUa as a comparison compressor from now on. The compression speed is the ratio of the uncompressed file size and the time needed for compression. Compared with LCQS and 7-zip, The compression speed of CMIC is better on all datasets, it improves the compression speed 0.63% ~ 1079.82%. Compared with Gzip, the maximum improvement is 390.47%. But it does not do well on five datasets (No.1, No.7, No.8, NO.9, No.11). In the worst case, CMIC is 86.69% slower than Gzip. However, the averaged compression performance of Gzip is the worst among all six compressors. Compared with FCLQC, CMIC is 20 ~ 60 times slower than it. But the compression is better than FCLQC.

**Table 3:** Comparison results of compression speed

Dataset	Compression speed (MB/S)					CMIC accelerating ratio versus%			
	CMIC	LCQS	FCLQC	7-zip	Gzip	LCQS	FCLQC	7-zip	Gzip
1	5.592	5.073		1.511	**6.399**	10.23%	–	270.09%	− 12.61%
2	8.796	8.741	**303.66**	6.435	4.523	0.63%	− 3352.25%	36.69%	94.47%
3	10.556	9.472	**329.16**	1.213	2.292	11.44%	− 3018.23%	770.24%	360.56%
4	12.86	11.074	**290.23**	1.09	2.622	16.13%	− 2156.84%	1079.82%	390.47%
5	**8.406**	6.981	–	2.822	2.0538	20.41%	–	197.87%	309.29%
6	**8.012**	6.983	–	2.846	2.768	14.74%	–	181.52%	189.45%
7	2.831	2.787	**159.12**	0.986	21.274	1.58%	− 5520.63%	187.12%	− 86.69%
8	3.532	3.374	**265,29**	1.882	9.876	4.68%	–	87.67%	− 64.24%
9	6.621	6.126	**421.58**	3.063	13.644	8.08%	− 6267.32%	116.16%	− 51.47%
10	5.444	5.389	**312.74**	2.351	3.36	1.02%	− 5644.67%	131.56%	62.02%
11	6.095	5.274	–	2.586	**23.328**	15.57%	–	135.69%	− 73.87%

Bold denotes the fastest compression speed

Table 4 shows CMIC and LCQS decompression speed. The decompression speed is the number of the quality score lines that the compressor can decompress per second. However, neither Gzip nor 7-zip supports decompressing files by lines. Therefore, the decompression speed of CMIC is only compared with LCQS. CMIC performs well on all of the datasets. Compared with LCQS, CMIC has a maximum improvement of 32.92%.

**Table 4:** Comparison results of decompression speed

Dataset	Decompression speed (thousand lines/s)		CMIC accelerating ratio versus%
	CMIC	LCQS	
1	2.43	1.36	32.92%
2	2.51	2.31	7.97%
3	2.67	2.10	21.35%
4	2.42	2.31	4.55%
5	3.13	2.96	5.43%
6	3.21	2.85	11.21%
7	2.20	2.11	4.27%
8	2.67	2.16	19.10%
9	1.82	1.73	4.95%
10	2.77	2.75	0.72%
11	1.63	1.47	9.82%

Table 5 shows CMIC and LCQS random access speed. Here, the random access speed refers to the time required for locating the data blocks that contain the target quality score lines. The experiment design is as follows. We select No.3, No.4, No.9, No.10, and No. 11 datasets as our test data. Since they are larger than other datasets. We select some search ranges for target quality score lines as the test intervals. In each interval, 10,000 lines of data are randomly selected as the access targets. 100 experiments are conducted for each interval, and the averaged random access time is taken at last. It is obvious that CMIC has great advantages in the random access speed. Compared with LCQS, No.11 has achieved the best results. CMIC improves by 396.1%. The larger the size of the quality score file, the more improvement of the averaged random access time of CMIC, since the binary search is apparently superior than the linear search used in LCQS. About the size of the index used, because we use a light wight index building method, the index table takes up only a small amount of space. For example, for the No.4 dataset, the size of the index table accounts for 0.016% of the compressed file, for the No.11 dataset, the size of the index table accounts for 0.013% of the compressed file.

**Table 5:** Comparison results of random access speed

Methods	Random access speed (ms)				
	(No.3) [75200000–75600000]	(No.4)[300500000–300900000]	(No.9) [120400000–121000000]	(No.10) [4300000,4700000]	(No.11) [700000–1100000]
CMIC	**153.0**	**290.2**	**206.1**	**146.6**	**100.0**
LCQS	524.4	869.3	530.5	418.0	496.1

	CMIC accelerating ratio versus%				
	242.7%	199.6%	157.4%	185.1%	396.1%

Bold denotes the fastest random access speed

As we all know, when the size of the block is small, the compression efficiency will be reduced, and the decompression time by random access will be improved. On the contrary, the compression efficiency will be improved, and the decompression time by random access will slow down. Table 6 shows the decompression times by random access for different block sizes (The size of the block is the number of quality score lines). We select a line in the file (such as line 80,000) as our random access object. As can be seen from Table 6, when the block size is large, the decompression time will be significantly longer, and vice versa.

**Table 6:** The decompression times by random access for different block sizes

Dataset	The block size			
	15,000	20,000	25,000	30,000
1	149.5	201.2	242.8	298.6
3	136.8	176.3	225.6	274.5
4	145.2	194.7	235.7	289.2
10	140.8	186.4	230.3	284.6

About the maximum CPU memory usage. Because CMIC algorithm needs to work in parallel, the data is compressed needs to be stored in memory, and each thread needs to occupy the corresponding workspace. For example, for No.5 dataset, its file size is 798985944 bytes, the maximum CPU memory usage in the compression phase is 9.5 GB, the maximum CPU memory usage in the decompression phase is 13.5 GB, and the maximum running memory usage in the random access phase is 0.6976 GB. Especially for the No.4 dataset, its file size is 431333335476 bytes, and its maximum memory usage in the decompression stage is 51.5 GB. Therefore, the memory usage of CMIC depends on the size of compressed data.

## Conclusion

This paper presents CMIC, a quality score compressor that supports random access. Experiments show that CMIC has advantages in terms of the compression rate and the random access time over all 11 datasets. Although the compression speed is not as fast as Gzip on some datasets, the compression efficiency is much better than that of Gzip. The proposed quality score compressor can be integrated into other FASTQ file compressors to improve their overall compression performance. However, in order to improve the compression efficiency of base sequences, it is usually necessary to classify base sequences. This will be different from the classification of quality score lines in this algorithm. Therefore, some kind of index has to be built for the base sequence to support random access. About the future work, In the FASTQ file generated by NPS, there is a certain correlation between the base sequence and the quality score sequence of each read. We hope to improve the compression efficiency of the quality score by using this correlation while ensuring the random access performance.

About the future work, In the FASTQ file generated by NPS, there is a certain correlation between the base sequence and the quality score sequence of each read. We hope to improve the compression efficiency of the quality score by using this correlation while ensuring the random access performance.

## Availability and requirements

- Project name: CMIC.
- Project home page: https://github.com/Humonex/Cmic.
- Operating systems: Linux
- Programming language: C++
- Other requirements: gcc 5.4.0.
- License: The MIT License.
- Any restrictions to use by non-academics: For commercial use, please contact the authors.

## Supplementary Information

**Additional file 1**. The pseudocode of the mapping algorithm.

## Data Availability

This database is public. The datasets (ID: SRR1284073, SRR327342, SRR870667, ERR091571, SRR003187, SRR003177, SRR007215, SRR010712, SRR070253, SRR801793, SRR14340293) supporting the conclusions of this paper are publicly available from https://trace.ncbi.nlm.nih.gov/Traces/sra/.

## Abbreviations

MB: Megabyte
NGS: Next generation sequencing
NPS: Nanopore sequencing
SIMD: Single instruction multiple data
CABAC: Context-adaptive binary arithmetic coder
