# Supplementary material for: CMIC: an efficient quality score compressor with random access functionality
Source: BMC Bioinformatics. 2022 Jul 23;23:294. doi: 10.1186/s12859-022-04837-1 (PMC9308261; doi:10.1186/s12859-022-04837-1)
Supplement: Supplementary file 1 — Additional file 1. The pseudocode of the mapping algorithm. [file 12859_2022_4837_MOESM1_ESM.pdf]

Algorithm 1: The mapping Algorithm

Input: The quality score line Q and The largest number of the quality score S.

Output: The quality score line after mapping processing.

while $(c = S.get())! = EOF$ **do**
// **Case 1**: **When adjacent quality scores are close to C**
if $Q[i] > S \; and \; S \le Q[i] \le S + 3 \; and \; S \le Q[i+1] \le S + 3$ **then**
  if $S \le Q[i+2] \le S + 3$ **then**
    $Q[i] \leftarrow 319 + ((S+3) - Q[i+2]) + 4 * ((S+3) - Q[i+1]) + 16 * ((S+3) - Q[i]);$
  else
    $Q[i] \leftarrow 255 + ((S+7) - Q[i+1]) + 8 * ((S+7) - Q[i])$
  end if
else if $Q[i] > S \; and \; S \le Q[i] \le S + 3 \; and \; S \le Q[i+1] \le S + 7 \; then$
    $Q[i] \leftarrow 255 + ((S+7) - Q[i+1]) + 8 * ((S+7) - Q[i])$
end if

// **Case 2: When adjacent quality scores are slightly far away from C**
if $Q[i] > S \; and \; S \le Q[i] \le S + 7 \; and \; S \le Q[i+1] \le S + 7$ **then**
    $Q[i] \leftarrow 255 + ((S+7) - Q[i+1]) + 8 * ((S+7) - Q[i])$
else if $Q[i] < S \; and \; S - 3 \le Q[i] \le S - 3 \le Q[i+1] \le S$ **then**
  if $S - 3 \le Q[i+2] \le S$ **then**
    $Q[i] \leftarrow ((Q[i] - (S-3)) * 16 + (Q[i+1] - (S-3)) * 4 + (Q[i+2] - (S-3) + 137);$
  else
    $Q[i] \leftarrow ((Q[i] - (S-7)) * 8 + (Q[i+1] - (S-7) + 73);$
  end if
else if $Q[i] < S \; and \; S - 3 \le Q[i] \le S \; and \; S - 7 \le Q[i+1] \le S$ **then**
    $Q[i] \leftarrow ((Q[i] - (S-7)) * 8 + (Q[i+1] - (S-7) + 73);$
end if

// **Case 3: When adjacent quality scores are equal to C**
if $Q[i] = S$ **then**
    $Q[i] \leftarrow 199 + len$ // len means the number of quality scores are equal to C
end if

// **Case 4: When the current quality score is far from C**
    $Q[i] \leftarrow= Q[i] - 32;$
end while
